# A scoring system for AML patients aged 70 years or older, eligible for intensive chemotherapy: a study based on a large European data set using the DATAML, SAL, and PETHEMA registries

**DOI:** 10.1038/s41408-022-00700-x

**Published:** 2022-07-11

**Authors:** Emilie Bérard, Christoph Röllig, Sarah Bertoli, Arnaud Pigneux, Suzanne Tavitian, Michael Kramer, Hubert Serve, Martin Bornhäuser, Uwe Platzbecker, Carsten Müller-Tidow, Claudia D. Baldus, David Martínez-Cuadrón, Josefina Serrano, Pilar Martínez-Sánchez, Eduardo Rodríguez Arbolí, Cristina Gil, Juan Bergua, Teresa Bernal, Adolfo de la Fuente Burguera, Eric Delabesse, Audrey Bidet, Pierre-Yves Dumas, Pau Montesinos, Christian Récher

**Affiliations:** 1 Centre Hospitalier Universitaire de Toulouse, Service d’Epidémiologie, CERPOP, Inserm, Université Toulouse III Paul Sabatier, Toulouse, France; 2 Medizinische Klinik und Poliklinik I, Universitätsklinikum TU Dresden, Dresden, Germany; 3 Centre Hospitalier Universitaire de Toulouse, Institut Universitaire du Cancer de Toulouse Oncopole, Université Toulouse III Paul Sabatier, Toulouse, France; 4 CHU Bordeaux, Service d’Hématologie Clinique et de Thérapie Cellulaire, Université de Bordeaux, Institut National de la Santé et de la Recherche Médicale, U1035, 33000 Bordeaux, France; 5 Medizinische Klinik 2, Universitätsklinikum Frankfurt, Frankfurt/Main, Germany; 6 Klinik und Poliklinik für Hämatologie, Zelltherapie und Hämostaseologie, Universitätsklinikum Leipzig, Leipzig, Germany; 7 Klinik für Hämatologie, Onkologie und Rheumatologie, Universitätsklinikum Heidelberg, Heidelberg, Germany; 8 Klinik für Innere Medizin II, Universitätsklinikum Schleswig-Holstein, Kiel, Germany; 9 Hospital Universitari i Politècnic La Fe, Valencia, Spain; Instituto de Investigación Sanitaria La Fe (IISLAFE), Valencia, Spain; 10 Hospital Universitario Reina Sofía-IMIBIC, Córdoba, Spain; 11 Hospital Universitario 12 de Octubre, Madrid, Spain; 12 Hospital Universitario Virgen del Rocío, Sevilla, Spain; 13 Hospital General Universitario de Alicante, Alicante, Spain; 14 Hospital San Pedro Alcántara, Cáceres, Spain; 15 Hospital Universitario Central de Asturias, Asturias, Spain; 16 MD Anderson Cancer Center Madrid, Madrid, Spain; 17 Centre Hospitalier Universitaire de Toulouse, Institut Universitaire du Cancer de Toulouse Oncopole, Laboratoire d’Hématologie Biologique, Toulouse, France; 18 CHU Bordeaux, Laboratoire d’Hématologie Biologique, F-33000 Bordeaux, France

**Keywords:** Acute myeloid leukaemia, Risk factors

## Abstract

In a context of therapeutic revolution in older adults with AML, it is becoming increasingly important to select patients for the various treatment options by taking account of short-term efficacy and toxicity as well as long-term survival. Here, the data from three European registries for 1,199 AML patients aged 70 years or older treated with intensive chemotherapy were used to develop a prognostic scoring system. The median follow-up was 50.8 months. In the training set of 636 patients, age, performance status, secondary AML, leukocytosis, and cytogenetics, as well as *NPM1* mutations (without *FLT3*-ITD), were all significantly associated with overall survival, albeit not to the same degree. These factors were used to develop a score that predicts long-term overall survival. Three risk-groups were identified: a lower, intermediate and higher-risk score with predicted 5-year overall survival (OS) probabilities of ≥12% (*n* = 283, 51%; median OS = 18 months), 3–12% (*n* = 226, 41%; median OS = 9 months) and <3% (*n* = 47, 8%; median OS = 3 months), respectively. This scoring system was also significantly associated with complete remission, early death and relapse-free survival; performed similarly in the external validation cohort (*n* = 563) and showed a lower false-positive rate than previously published scores. The European Scoring System ≥70, easy for routine calculation, predicts long-term survival in older AML patients considered for intensive chemotherapy.

## Introduction

With a median age of approximately 70 years at diagnosis, acute myeloid leukemia (AML) is a disease of the elderly. AML patients ≥70 years of age have a worse prognosis than younger patients both because of the accumulation of comorbidities that increase the risk of treatment toxicity and because of the unfavorable biological characteristics of the disease which increase the risk of treatment failure [1].

To date, intensive chemotherapy (IC) and hypomethylating agents (HMAs) or low-dose cytarabine combined with the Bcl2 inhibitor venetoclax are the main standard treatment options in these patients although venetoclax is not yet fully approved or reimbursed in some countries [2]. Although the drug-label for venetoclax and low-intensity therapy is limited to patients deemed ineligible for IC, there is a significant number of patients who can be selected for either of these two therapeutic strategies in daily practice, particularly those ≥70 years old. In fact, recent clinical trials have demonstrated that the addition of venetoclax to low intensity therapy in patients unfit for IC has resulted in remission rates and median overall survival approaching that of IC in fitter patients [3, 4]. Therefore, there is an increasing number of physicians who are tempted to offer venetoclax and low-intensity treatment rather than IC in older fit AML patients [5–7].

The overall results of IC remains largely unsatisfactory in this setting [8]. However, we have recently shown that IC offers higher chances of complete remission and better long-term survival compared to HMAs despite a higher rate of early toxicity in a series of 2,272 patients ≥70 years old [9]. Furthermore, it is conceivable that outcomes with IC may improve significantly with the advent of recently approved drugs that may limit early toxicity and increase remission rate, such as the dual-drug liposomal combination of daunorubicin and cytarabine CPX-351, or prolong response and improve overall survival, such as oral azacitidine used as maintenance therapy in patients in complete remission after IC [10, 11]. Therefore, it is of upmost importance to select patients who can significantly benefit from IC in terms of long-term survival. Over the past decade, a series of prognostic scores have been built to determine which patients might benefit most from IC in terms of early mortality, remission, and survival. Most of these scoring systems were based on factors related to patients (age, performance status, comorbidity index), disease history (history of hematological disorders or cytotoxic therapy), and initial disease characteristics (proliferation markers such as leukocytosis or lactate dehydrogenase, cytogenetic risk, platelet count) [12–16]. Few of them have included molecular markers [17, 18].

Our primary aim was to build and assess the validity of a European scoring system for long-term overall survival in AML patients ≥70 years old (ESS70+) who were selected routinely for IC using parameters available at diagnosis [19]. We then compared the validity of our ESS70+ with previously published scoring systems for older patients treated with IC.

## Subjects and method

### Patients

In the previous paper, all patients ≥70 years old with newly diagnosed AML (excluding acute promyelocytic leukemia) between 01/01/2007 and 30/06/2018 (*n* = 3,700) were included in a database established from the French Toulouse-Bordeaux DATAML (2 tertiary centers and 21 secondary centers), German Study Alliance Leukemia (SAL, 46 centers) and Programa Español de Tratamientos en Hematología (PETHEMA, 88 centers) registries whatever their treatment (best supportive care, low-dose cytarabine, semi-intensive regimen, HMA or IC). The total number of AML patients ≥70 years old registered during this 11.5-year period of time was 4,652 [9]. The present study designed to construct a prognostic score included patients whose first line treatment was IC (mainly standard 3 + 7 which combines daunorubicin and cytarabine or idarubicin and cytarabine with or without lomustine, *n* = 1,199) [9]. A data set was collected for each patient, including age, gender, date of diagnosis, AML status (de novo or secondary), ECOG performance status, white blood cell count, percentage of peripheral and bone marrow blasts, LDH, cytogenetic risk, *NPM1*, *FLT3*-ITD, *CEBPA*, *IDH1*, *IDH2, TP53* mutational status at diagnosis, response to treatment, allogeneic hematopoietic stem cell transplantation in first complete remission, date of relapse and/or death.

This study was conducted in accordance with the Declaration of Helsinki. All registries were approved by institutional review boards or national authorities, and informed consent was obtained from all patients.

### Endpoints

Response to treatment (complete remission, CR), early-death (ED: day-30 and day-60 death), relapse, relapse-free survival (RFS), and overall survival (OS) were defined according to the European Leukemia Net (ELN) criteria [20].

### Statistical analysis

Data from the DATAML and PETHEMA registries (*N* = 636) were used as a training set and data from the SAL registry (*N* = 563) were used as an external validation set. The scoring system was based on OS (as the time between diagnosis and death or the last contact) censored at 5 years and included 6 candidate predictors (age, ECOG performance status (PS), white blood cell count (WBC) at diagnosis, secondary vs de novo AML, cytogenetic risk and NPM1/FLT3-ITD mutations) [9]. According to guidelines, missing values were imputed using multiple imputations in the training set [21]. After multiple imputation (for PS, WBC at diagnosis, and secondary vs de novo AML), a multivariate Cox proportional hazards model was used to assess β-coefficients of the survival predictors. Then, a linear predictor (LP) based on the β-coefficients was computed for all patients with a complete case in the training set. Moreover, to provide a simple tool for clinical practice, we developed score sheets using the formula (β-coefficient/abs(lowest β-coefficient)) rounded off to the nearest integer. Based on the predicted 5-year overall survival probability (S(t/LP) = S0(t)exp(β.LP)), three risk score categories were created according to previously published survival probabilities from European data on DATAML, PETHEMA, and SAL registries for IC (12%) and HMA (3%) [9]. As recommended, to verify the internal validity of the LP, the R²D described by Royston and Sauerbrei (that is a measure of explained variation for survival models) was assessed together with measures of calibration and discrimination, in the training cohort [21]. Performance for discriminating patients who died from those who survived was assessed using Harrell’s concordance index (C-index). The C-index uses values from 0.5 (no discrimination) to 1.0 (perfect discrimination). Discrimination was also assessed using Kaplan–Meier survival curves for the risk groups and estimating hazard ratios along with their 95% confidence interval (CI). Finally, discrimination was verified by assessing the effect of risk groups on other endpoints (CR, day-30 and day-60 death, and RFS). To verify the external validity, the R²D and C-index (for Cox model with the risk groups as factor) together with Kaplan–Meier survival curves for the risk groups were assessed in the external validation set. Finally, in the validation set, we compared the predictive performance of our risk groups to published prognostic indices. Tests were two-sided and *P*-values lower than 0.05 were considered significant. Statistical analyses were performed using STATA statistical software, version 17.0 (STATA Corp., College Station, TX). See Supplementary Material online for detailed statistical analyses.

## Results

### Patients’ characteristics

The study included 1,199 European patients, diagnosed between 2007 and 2018, treated with IC (56% were men). The median age was 74 years [inter-quartile range (IQR): 72–76] and 75% of the patients presented de novo AML, ECOG PS ≤ 1, and intermediate cytogenetic risk. *NPM1* and *FLT3*-ITD mutations were detected in 306 (35.6%) and 172 (19.8%) patients, respectively. All patients’ characteristics are described in Supplementary Table 1. Complete remission or complete remission with incomplete hematologic recovery was 56.1% whereas day-30 and day-60 mortality were 13.0% and 20.6%, respectively, and the median overall survival was 10.9 months (95%CI: 9.7–11.6) (median follow-up, 50.8 months). Of note, OS of patients aged 75–79 years was not significantly different from that of patients aged 70–74 years (Hazard Ratio, 1.05, 95%CI:0.85–1.30; *p* = 0.622).

### Development of a new European scoring system (ESS70+) using the training set

**Fig. 1:**
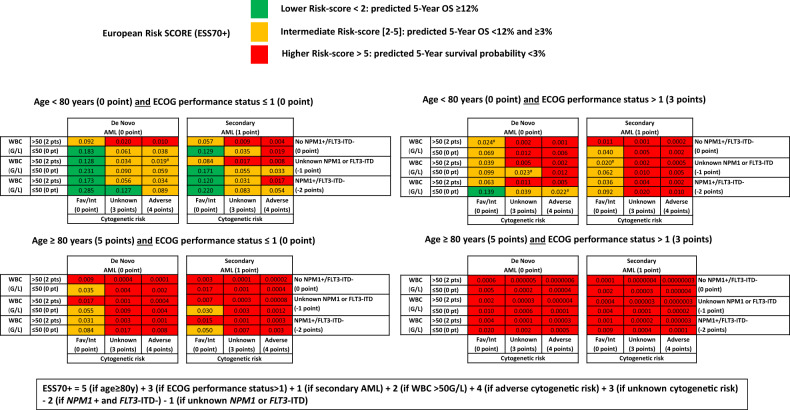
Predicted 5-year overall survival probability* using the Linear Predictor (LP). For example, for a patient aged 75 years (i.e., <80 y), with ECOG performance status>1, secondary AML, WBC ≤ 50 G/L, favorable cytogenetic risk and unknown *NPM1* or *FLT3*-ITD mutation, ESS70 + score was equal to 0 + 3 + 1 + 0 + 0 – 1 = 3 and predicted 5-year overall probability was equal to 0.062. ECOG Eastern Cooperative Oncology Group, WBC white blood cell count, *NPM1*+ *NPM1* mutation, fav favorable, Int intermediate, pt(s), point(s). *S(t/LP) = S0(t)exp(β.LP) where S0(t) is the survival function of the baseline population with LP = 0 (i.e., the 5-year survival probability of the population having a LP = 0), called the baseline survival function and equal to 0.183 in the complete cases training set; β = 0.965; LP = 0.71 (if age≥80 y) + 0.47 (if ECOG performance status>1) + 0.68 (if adverse cytogenetic risk) + 0.51 (if unknown cytogenetic risk) + 0.35 (if WBC > 50 G/L) + 0.19 (if secondary AML) − 0.31 (if *NPM1*+ and *FLT3*-ITD−) − 0.15 (if unknown *NPM1* or *FLT3*-ITD). ^#^Discrepancies between predicted 5-year overall survival probability and ESS70+ were due to rounded off to the nearest integer of each point (β-coefficient/abs(lowest β-coefficient)) of ESS70+.

After multiple imputation of missing data in PS, WBC at diagnosis, and secondary vs de novo AML, all the chosen predictors (age, performance status, WBC at diagnosis, secondary vs de novo AML, cytogenetic risk, and *NPM1*/*FLT3*-ITD mutations) were included in a multivariate Cox proportional hazard model that predicts OS (Table 1). It is of note that in the complete cases training set (before multiple imputation) results were not significantly different. The parameters (β) were used to compute for each individual (of the complete cases training set (*N* = 556)) a risk score called the Linear Predictor (LP) of death risk (Table 1). A high LP score reflects a worse prognosis while a low LP score represents a better prognosis. We then computed the predicted survival probability at 5 years for each patient using LP (Fig. 1). To provide a tool, easy to use in clinical practice, score sheets (ESS70 + ) were developed based on β-coefficients (Table 1). A high score reflects a poor prognosis and a low score a better prognosis. Accordingly, three categories of risks were created using expected survival probabilities previously published from European data on DATAML, PETHEMA and SAL registries for patients treated with IC (12%) or HMA (3%) [9]: lower-risk score (<2): predicted 5-year survival probability ≥12%, *n* = 283 (51%); intermediate-risk score: (2−5) predicted 5-year survival probability <12% and ≥3%, *n* = 226 (41%); higher-risk score (>5): predicted 5-year survival probability <3%, *n* = 47 (8%). All predicted 5-year survival probabilities using the Linear Predictor are detailed in Fig. 1.

**Table 1:** Overall Survival prognostic model in the training set (*N* = 636; 15 imputations)—Multivariate Cox proportional hazard model.

Prognostic factors	β (95% CI)	HR (95% CI)	*P*-value
Age ≥ 80 years	0.71 (0.23; 1.19)	2.03 (1.26; 3.28)	0.004
ECOG performance status>1	0.47 (0.26; 0.69)	1.60 (1.29; 1.99)	<0.001
Adverse cytogenetic risk	0.68 (0.44; 0.92)	1.98 (1.56; 2.51)	<0.001
Unknown cytogenetic risk	0.51 (0.21; 0.82)	1.67 (1.24; 2.26)	0.001
WBC > 50 Giga/L	0.35 (0.14; 0.56)	1.42 (1.15; 1.75)	0.001
Secondary AML	0.19 (−0.03 ;0.41)	1.21 (0.97; 1.51)	0.086
*NPM1*^+^, *FLT3*-ITD^−^	−0.31 (−0.60; −0.03)	0.73 (0.55; 0.97)	0.031
Unknown *NPM1* or *FLT3*-ITD mutation	−0.15 (−0.35; 0.05)	0.86 (0.70; 1.05)	0.137

LP = 0.71 (if age ≥ 80 y) + 0.47 (if PS ≥ 2) + 0.68 (if adverse cytogenetic risk) + 0.51 (if missing cytogenetic risk) + 0.35 (if WBC > 50 G/L) + 0.19 (if secondary AML) −0.31 (if *NPM1* mutation and no *FLT3*^-^ITD mutation) − 0.15 (if missing *NPM1* or *FLT3*-ITD).

ESS70+ =5^#^ (if age ≥ 80 y) + 3 (if PS ≥ 2) + 4 (if adverse cytogenetic risk) + 3 (if missing cytogenetic risk) + 2 (if WBC > 50 G/L) + 1 (if secondary AML) − 2 (if *NPM1* mutations and no *FLT3*-ITD mutation) − 1 (if missing *NPM1* or *FLT3*-ITD).

^#^(β-coefficient/abs(lowest β-coefficient)) rounded off to the nearest integer.

*CI* Confidence Interval, *HR* Hazard Ratio, *WBC* White Blood Cell Count, *LP* Linear Predictor of death risk, *PS* ECOG Performance Status, *ESS* European scoring system.

### Calibration and discrimination assessment using the training set

In the complete cases training set (*n* = 556), using the continuous LP, the calibration slope (β-coefficient) was not significantly different from 1, indicating good calibration (Supplementary Fig. 1A). Moreover, a graphical assessment of calibration was done with predicted 5-year probabilities on the x-axis and the observed outcome on the y-axis (Supplementary Fig. 1B). Predictions were close to the 45° line suggesting no major calibration issue in the training set. The R²D (a measure of explained variation for survival models) was equal to 9% [95%CI = 5–14] for the Cox model with the LP as the factor and the C-index (a measure of performance for discriminating patients who died from those who survived) after optimism correction was equal to 62% [95%CI = 59–65].

**Fig. 2:**
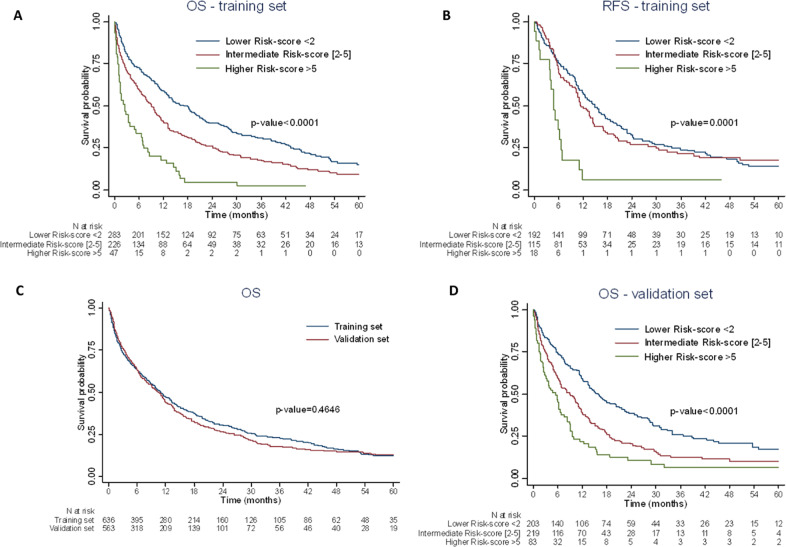
Kaplan-Meier survival curves. **A** OS Kaplan–Meier survival curves according to the ESS70+ risk categories at up to 5 years (Training set complete cases). **B** RFS Kaplan–Meier survival curves according to the ESS70+ risk categories at up to 5 years (Training set complete cases). **C** OS Kaplan–Meier survival curves at up to 5 years for the training and validation sets. **D** OS Kaplan–Meier survival curves according to the ESS70+ risk categories at up to 5 years (validation set complete cases).

Discrimination was also explored through Kaplan-Meier curves and HR estimates for risk groups to assess the distance between the curves for the lower, intermediate, and higher-risk groups (Table 2). The risk categories were significantly associated with OS (*p* < 0.0001). Kaplan–Meier curves for the 3 risk categories are presented in Fig. 2A. We observed a large distance between the 3 curves which confirms the difference in the death risk associated with each of the 3 risk categories of the prognostic model (*p* < 0.0001). Indeed, median OS was 18 months (IQR: 4–43) for lower-risk score, 9 months (IQR 2−24) for intermediate-risk score and 3 months (1–7) for higher- risk score.

**Table 2:** Discrimination assessment for the ESS70+ in 3 risk categories using the training set (*N* = 556).

	Overall Survival (OS)		
	Hazard Ratio (HR) estimate	95% Confidence Interval (CI) of HR	*P*-value
**Risk category**			
Lower-risk score <2	1.00		
Intermediate-risk score [2–5]	1.48	[1.22; 1.80]	<0.001
Higher-risk score >5	3.33	[2.40; 4.63]	<0.001
	**Relapse Free Survival (*N*** = **325)**		
	**Hazard Ratio (HR) estimate**	**95% CI of HR**	***P*-value**
**Risk category**			
Lower-risk score <2	1.00		
Intermediate-risk score (2–5)	1.07	[0.82; 1.40]	0.600
Higher-risk score >5	3.04	[1.80; 5.14]	<0.001
	**Complete Remission**		
	**No**	**Yes**	***P*-value**
**Risk category**			<0.0001
Lower-risk score <2, *n* (%)	91 (32.2)	192 (67.8)	
Intermediate-risk score [2–5], *n* (%)	111 (49.1)	115 (50.9)	
Higher-risk score >5, *n* (%)	29 (61.7)	18 (38.3)	
	**Day-30 death**		
	**No**	**Yes**	***P*-value**
**Risk category**			<0.0001
Lower-risk score <2, *n* (%)	256 (90.5)	27 (9.5)	
Intermediate-risk score [2–5], *n* (%)	186 (82.3)	40 (17.7)	
Higher-risk score >5, *n* (%)	31 (66.0)	16 (34.0)	
	**Day-60 death**		
	**No**	**Yes**	***P*-value**
**Risk category**			<0.0001
Lower-risk score <2, *n* (%)	240 (84.8)	43 (15.2)	
Intermediate-risk score [2–5], *n* (%)	172 (76.1)	54 (23.9)	
Higher-risk score >5, *n* (%)	25 (53.2)	22 (46.8)

Finally, discrimination was checked by assessing the effect of risk groups on other endpoints (CR, ED, and RFS). The risk categories were significantly associated to other endpoints (Table 2). RFS Kaplan–Meier curves for the 3 risk categories are presented in Fig. 2B. We observed a large distance between the higher-risk category vs lower- or intermediate-risk category, indicating good discrimination (*p* = 0.0001).

### External validation of the new ESS70+using a validation set

Survival data and characteristics of the European scoring system in the training and validation sets are described in Table 3 and Fig. 2C. The OS Kaplan–Meier survival curve was not significantly different in the validation set compared to the training dataset (*p* = 0.4646). The LP score tended to be higher in the validation set compared to the training dataset. In fact, patients were older and more frequently had secondary AML in the validation set (Supplementary Table 1) and were, therefore, more at risk due to their profile. Accordingly, there were more higher-risk patients in the validation dataset compared to the training set and fewer lower-risk patients. The C-Index (and R²D) for the Cox model with the 3 risk categories was the same in the validation set and in the training dataset, indicating the same discrimination ability (and adequacy for data). Moreover, in the validation set, OS Kaplan–Meier survival curves showed a clear separation between the 3 risk groups, as observed in the training dataset which indicates good discrimination (Fig. 2D). A good discrimination was also observed for CR and ED (Table 3).

**Table 3:** Survival data and characteristics of the ESS70+ in the training and validation sets.

	Training set (Complete cases; *N* = 556)	Validation set (Complete cases; *N* = 505)
Median FU (month) [IQR]	58 [33–60]	46 [19–60]
Median OS (month) [IQR]	11 [3–31]	10 [3–26]
KM 5-Year Survival probability [95%CI]	0.123 [0.095–0.156]	0.128 [0.094–0.166]
S0 at 5 years (Cox OS with LP = 0)	0.183	0.194
Mean LP (SD)	0.32 (0.43)	0.48 (0.49)
Mean SCORE (SD)	2 (3)	3 (3)
**Risk categories**	***N*** **(%)**	***N*** **(%)**
Lower risk <2	283 (51)	203 (40)
Intermediate risk [2–5]	226 (41)	219 (43)
Higher risk >5	47 (8)	83 (16)
**Risk categories**	**Median OS (month) [IQR]**	**Median OS (month) [IQR]**
Lower risk <2	18 [4–43]	16 [6–39]
Intermediate risk [2–5]	9 [2–24]	9 [3–19]
Higher risk >5	3 [1–7]	5 [2–10]
**Risk categories**	**KM 5-Year Survival probability [95%CI]**	**KM 5-Year Survival probability [95%CI]**
Lower risk <2	0.15 [0.10–0.21]	0.17 [0.11–0.24]
Intermediate risk [2–5]	0.09 [0.06–0.14]	0.10 [0.06–0.17]
Higher risk >5	Not observed (0.02 [0.00–0.10] at 42 month)	0.06 [0.02–0.15]
**Risk categories** [Lower/Intermediate/Higher]	**C-Index**	**C-Index**
	0.59 [0.56–0.62]	0.59 [0.57–0.62]
**Risk categories** [Lower/Intermediate /Higher]	**R²D**	**R²D**
	0.08 [0.04–0.13]	0.07 [0.03–0.12]
**Risk categories**	**Complete Remission,** ***n*** **(%)**	**Complete Remission,** ***n*** **(%)**
Lower risk <2	192 (67.8)	144 (70.9)
Intermediate risk [2–5]	115 (50.9)	105 (47.9)
Higher risk >5	18 (38.3)	25 (30.1)
**Risk categories**	**Day-30 death,** ***n*** **(%)**	**Day-30 death,** ***n*** **(%)**
Lower risk <2	27 (9.5)	17 (8.4)
Intermediate risk [2–5]	40 (17.7)	20 (9.1)
Higher risk >5	16 (34.0)	15 (18.1)
**Risk categories**	**Day-60 death,** ***n*** **(%)**	**Day-60 death,** ***n*** **(%)**
Lower risk <2	43 (15.2)	24 (11.8)
Intermediate risk [2–5]	54 (23.9)	46 (21.0)
Higher risk >5	22 (46.8)	25 (30.1)

*FU* Follow-up, *OS* Overall Survival, *KM* Kaplan–Meier, *LP* Linear Predictor, S0 at 5 years: 5-year survival probability of population having a LP = 0; *SCORE* the new European scoring system, *SD* Standard Deviation, *IQR* Interquartile Range.

### Comparison of the predictive performances of the ESS70+ versus published prognosis scores using the validation set

We chose to compare the ESS70+ with the ALFA and MRC scores because our data were applicable to these scoring systems contrary to other scores that contained variables not collected in our registries [15, 16]. The different risk scores were significantly associated with OS in the validation dataset (Table 4). The C-Index (and R²D) was not significantly different for the ESS70+ in 3 categories compared to ALFA or MRC prognostic indices indicating the same discrimination ability (and adequacy for data) [15, 16]. However, the false positive rate (FPR), which estimates the rate of patients identified as higher risk in the subset of those who survived, was significantly lower in the ESS70+ (FPR, 12% [95%CI: 7–19]) compared to ALFA (FPR, 38% [95%CI: 30–47]) or MRC (FPR, 64% [95%CI: 55–72]) prognostic scores.

**Table 4:** Comparison of the predictive performances of the ESS70+ versus published prognostic scores using the validation set.

Cox model for Overall Survival (*N* = 505)	Hazard Ratio [95% CI] (*P*-value)	C-Index [95% CI]	R²D [95% CI]	FPR [95% CI]
**M1: ESS70**+		0.59 [0.57–0.62]	0.07 [0.03–0.12]	16/133 = 12% [7–19]
Intermediate risk [2–5]	1.59 [1.27–2.00] (*p* < 0.001)			
Higher risk >5	2.42 [1.80–3.26] (*p* < 0.001)			
**M2: ALFA**^a^ **decisional index**	1.87 [1.52–2.30] (*p* < 0.001)	0.59 [0.56–0.62]	0.09 [0.04–0.14]	51/134 = 38% [30–47]
**M3: MRC**^b^ **prognostic index**		0.57 [0.54–0.59]	0.07 [0.03–0.13]	85/133 = 64% [55–72]
Standard risk	2.71 [1.10–6.67] (*p* = 0.031)			
Poor risk	4.27 [1.76–10.36] (*p* = 0.001)			

^a^ALFA decisional index 1+ vs 0 (15).

^b^MRC prognostic index: Standard risk or Poor risk vs Good risk (16).

*CI* Confidence Interval, *FPR* False positive rate (rate of patients identified as high risk in the subset of those who survived).

### Distribution of treatments in AML patients ≥70 years old

**Fig. 3:**
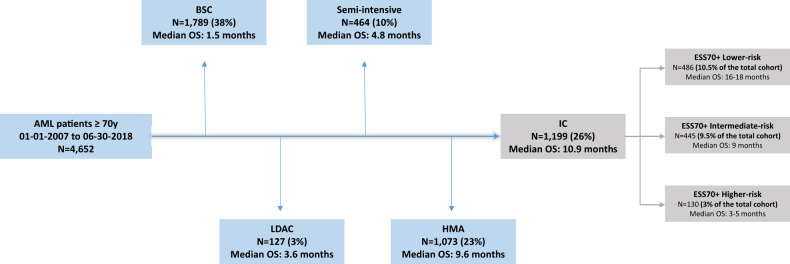
Distribution of first-line treatments in the total cohort of AML patients ≥70 y.

During the 11.5-year period of the study, 4652 patients were registered and their first-line treatment was BSC (38%), LDAC (3%), semi-intensive regimen (10%), HMA (23%) or IC (26%). Therefore, the proportion of patients with ESS70+ lower, intermediate or higher risk was 10.5%, 9.5% and 3% of the total cohort respectively (Fig. 3).

## Discussion

In this study, we specifically established a simple scoring system for key clinical endpoints, including long-term survival, in AML patients ≥70 y selected in real world for IC. Not surprisingly, we found that age, performance status, secondary AML, leukocytosis and cytogenetics, albeit not all to the same degree, were significantly associated with OS and similar to other scores. Interestingly, we confirmed the impact of *NPM1* mutations (without *FLT3*-ITD) as a favorable factor that should be taken account of when choosing first line treatment in older AML patients [17, 22–24].

We acknowledge that our ESS70+ does not have superior predictive abilities to previous comparable scores [15, 16]. However, ESS70+ appears to substantially reduce the false-positive rate thereby decreasing the risk of loss of chance related to non-choice of the IC as first line treatment using previous scores. Overall, with a performance for discriminating patients who died from those who survived (C-index) of approximately 60%, the predictive ability of these scores remains perfectible. A recent AML-composite model for 1-year mortality combining the hematopoietic cell transplantation–comorbidity index, age, and cytogenetic/molecular risks yielded a better C-statistic but remained <80% [18]. In our study, HCT-CI data were not fully collected to assess the relative weight of comorbidities in the score. However, in 856 patients from the DATAML and SAL registries, the median HCT-CI was 1 (IQR, 0-2) suggesting that comorbidities were taken account of by physicians before selecting the IC in most patients and that these variables are therefore unlikely to refine the score. Furthermore, the ESS70+ identified only 8% as higher-risk patients, which was probably due to the initial selection. In fact, patients at an advanced age (>80 y) with adverse-risk cytogenetics or a poor performance status were often not offered high-intensity chemotherapy in the centers that contributed to this registry.

Nevertheless, our study had several strengths that should be mentioned. First, the ESS70+ is an updated scoring system based on AML patients treated recently with lower false positive rate, from a large European cohort with external validation in other European patients (who were at a higher risk). In fact, even though the ESS70+ was higher in the German validation cohort, it retained good levels of prognostic and discriminative abilities. These findings validate the transportability to AML patients in other settings. In addition, our ESS70+ was developed based on patients selected for IC mostly outside of clinical trials, allowing application in daily practice.

The value of IC after 70 years of age remains a matter of debate [13]. Our registry allowed us to describe the therapeutic panorama chosen by physicians from 3 European countries. We have shown that a small proportion of these patients can still benefit from IC. It is very likely that the combinations of lower intensity treatments with bcl2 inhibitors will have similar or even better results although long-term survival data are lacking with these new therapies. Prospective clinical trials are warranted to determine whether IC can be definitively abandoned in this specific setting.

The median age of patients included in the ESS70+ was 74 years old. Therefore, many patients who were selected for IC may now have an indication to receive a hypomethylating agent plus venetoclax combination since this regimen was recently approved for patients 75 years or older regardless of other fitness parameters [5]. Whether the ESS70+ is relevant for patients treated with this novel standard of care or helps to select patients for one of the two strategies remains to be determined in future studies.

In conclusion, the ESS70+, based on a large population of older AML patients, is a score that is easy to calculate routinely with basic clinical and molecular parameters, so that long-term survival in older patients in whom intensive chemotherapy is being considered can be predicted.

### Reporting summary

Further information on research design is available in the Nature Research Reporting Summary linked to this article.

## Supplementary information

Supplementary Figure 1

Supplementary Table 1 Clean

Supplementary Material

Reporting Summary

## Data Availability

The datasets supporting the results presented in this article could be available to researchers who provide a methodologically sound proposal. The data will be provided after its de-identification, in compliance with applicable privacy laws, data protection, and requirements for consent and anonymization.
