# Supplementary material for: A scoring system for AML patients aged 70 years or older, eligible for intensive chemotherapy: a study based on a large European data set using the DATAML, SAL, and PETHEMA registries
Source: Blood Cancer J. 2022 Jul 11;12(7):107. doi: 10.1038/s41408-022-00700-x (PMC9276717; doi:10.1038/s41408-022-00700-x)
Supplement: Supplementary file 1 — Supplementary Figure 1 [file 41408_2022_700_MOESM1_ESM.docx]

**Supplementary Figure 1: Calibration assessment for the continuous linear predictor (LP) using training set**

1. Cox model at up to 5 years of follow-up with linear predictor as factor in complete cases training set (N=556)
2. Comparison of observed 5-Years survival probability according to Kaplan-Meier and 5-Years survival probability predicted by the Cox model with LP (N=556)

**A**

|  | **Factor** | **Parameter estimate β** | **95% CI of β** | **Hazard Ratio (HR) estimate** | **95% CI of HR** | **P-value** |
| --- | --- | --- | --- | --- | --- | --- |
| Training set complete cases | LP | 0.965 | [0.740 ; 1.191] | 2.63 | [2.10 ; 3.29] | <.001 |

**B**

CI: Confidence Interval
