# Supplementary material for: A scoring system for AML patients aged 70 years or older, eligible for intensive chemotherapy: a study based on a large European data set using the DATAML, SAL, and PETHEMA registries
Source: Blood Cancer J. 2022 Jul 11;12(7):107. doi: 10.1038/s41408-022-00700-x (PMC9276717; doi:10.1038/s41408-022-00700-x)
Supplement: Supplementary file 2 — Supplementary Table 1 Clean [file 41408_2022_700_MOESM2_ESM.docx]

**Supplementary Table 1: Patients characteristics**

|  |  | |  |  |
| --- | --- | --- | --- | --- |
|  | **TRAINING SET** | **VALIDATION SET** | **P-value** | **Total** |
|  | 636 (53.0%) | 563 (47.0%) |  | 1199 (100.0%) |
| Sex, n(%) |  |  | .273 |  |
| Man | 364 (57.3) | 305 (54.2) |  | 669 (55.8) |
| Woman | 271 (42.7) | 258 (45.8) |  | 529 (44.2) |
| Age, diagnosis (years) |  |  |  |  |
| n /missing | 636/0 | 563/0 |  | 1199/0 |
| Mean (SD) | 73.89 (2.86) | 75.10 (3.85) | <.0001 | 74.46 (3.41) |
| Median | 73.41 | 74.00 |  | 73.99 |
| IQR | [71.50; 75.66] | [72.00; 77.00] |  | [72.00; 76.00] |
| Min;Max | 70.00; 86.38 | 71.00; 90.00 |  | 70.00; 90.00 |
| AML status, n(%) |  |  | <.0001 |  |
| De novo | 485 (79.9) | 363 (69.9) |  | 848 (75.3) |
| Secondary | 122 (20.1) | 156 (30.1) |  | 278 (24.7) |
| ECOG performance status, n(%) |  |  | .666 |  |
| 0-1 | 437 (75.5) | 409 (74.4) |  | 846 (74.9) |
| 2-3-4 | 142 (24.5) | 141 (25.6) |  | 283 (25.1) |
| WBC in Giga/L |  |  |  |  |
| n /missing | 625/11 | 555/8 |  | 1180/19 |
| Mean (SD) | 40.07 (63.81) | 39.86 (58.74) |  | 39.97 (61.45) |
| Median | 8.98 | 9.82 |  | 9.30 |
| IQR | [2.30; 48.10] | [2.23; 56.45] | .778 | [2.30; 54.00] |
| Min;Max | 0.20; 433.00 | 0.06; 351.10 |  | 0.06; 433.00 |
| % of blood blasts |  |  |  |  |
| n /missing | 521/115 | 496/67 |  | 1017/182 |
| Mean (SD) | 40.84 (34.40) | 36.92 (32.13) | .061 | 38.93 (33.35) |
| Median | 35.00 | 28.00 |  | 30.00 |
| IQR | [7.00; 75.00] | [6.00; 68.00] |  | [6.00; 71.00] |
| Min;Max | 0.00; 99.00 | 0.00; 99.00 |  | 0.00; 99.00 |
| LDH in UI/L |  |  |  |  |
| n /missing | 521/115 | 544/19 |  | 1065/134 |
| Mean (SD) | 804.34 (1122.89) | 632.99 (792.76) |  | 716.81 (971.75) |
| Median | 473.00 | 391.00 |  | 429.00 |
| IQR | [274.0; 796.0] | [249.0; 688.0] | .001 | [258.0; 736.0] |
| Min;Max | 106.0;12806.0 | 99.0; 9086.0 |  | 99.0;12806.0 |
| % of blasts in the bone marrow |  |  |  |  |
| n /missing | 559/77 | 495/68 |  | 1054/145 |
| Mean (SD) | 60.33 (25.54) | 59.77 (24.82) | .719 | 60.07 (25.20) |
| Median | 61.00 | 61.00 |  | 61.00 |
| IQR | [36.00; 84.00] | [40.00; 80.00] |  | [38.00; 82.00] |
| Min;Max | 0.00; 100.00 | 1.00; 100.00 |  | 0.00; 100.00 |
| Cytogenetic prognosis, n(%) |  |  | .246 |  |
| Favorable | 28 (4.9) | 19 (3.9) |  | 47 (4.4) |
| Intermediate | 437 (76.5) | 358 (73.7) |  | 795 (75.2) |
| Adverse | 106 (18.6) | 109 (22.4) |  | 215 (20.3) |
| ELN 2010, n(%) |  |  | <.0001 |  |
| Favorable | 68 (13.2) | 87 (17.9) |  | 155 (15.5) |
| Intermediate 1 | 134 (26.0) | 187 (38.6) |  | 321 (32.1) |
| Intermediate 2 | 175 (33.9) | 102 (21.0) |  | 277 (27.7) |
| Adverse | 139 (26.9) | 109 (22.5) |  | 248 (24.8) |
| FLT3-ITD mutation, n(%) |  |  | .008 |  |
| No | 315 (76.5) | 383 (83.6) |  | 698 (80.2) |
| Yes | 97 (23.5) | 75 (16.4) |  | 172 (19.8) |
| NPM1 mutation, n(%) |  |  | .079 |  |
| No | 251 (61.4) | 302 (67.1) |  | 553 (64.4) |
| Yes | 158 (38.6) | 148 (32.9) |  | 306 (35.6) |
| TP53 mutation, n(%) |  |  | 1 |  |
| No | 4 (100.0) | 23 (82.1) |  | 27 (84.4) |
| Yes | 0 (0.0) | 5 (17.9) |  | 5 (15.6) |
| Inclusion in a clinical trial, n(%) |  |  | .449 |  |
| No | 557 (87.6) | 501 (89.0) |  | 1058 (88.2) |
| Yes | 79 (12.4) | 62 (11.0) |  | 141 (11.8) |
| Type of chemotherapy, n(%) |  |  | <.0001 |  |
| DA | 7 (1.1) | 425 (75.5) |  | 432 (36.0) |
| IA | 373 (58.6) | 8 (1.4) |  | 381 (31.8) |
| IACCNU | 214 (33.6) | 0 (0.0) |  | 214 (17.8) |
| FLAG | 5 (0.8) | 1 (0.2) |  | 6 (0.5) |
| Other | 37 (5.8) | 129 (22.9) |  | 166 (13.8) |
| Allogeneic stem cell transplantation, n(%) |  |  | <.0001 |  |
| No | 636 (100.0) | 493 (87.6) |  | 1129 (94.2) |
| Yes | 0 (0.0) | 70 (12.4) |  | 70 (5.8) |
|  |  |  |  |  |

TRAINING SET = DATAML and PETHEMA Registries; VALIDATION SET = SAL Registry; SD: Standard Deviation ; IQR : Inter-Quartile Range; WBC : White Blood Cell Count; DA : daunorubicin-cytarabine; IA: idarubicin-cytarabine; IACCNU: idarubicin-cytarabine-lomustine; FLAG: fludarabine-cytarabine-GCSF.
