# Supplementary material for: A scoring system for AML patients aged 70 years or older, eligible for intensive chemotherapy: a study based on a large European data set using the DATAML, SAL, and PETHEMA registries
Source: Blood Cancer J. 2022 Jul 11;12(7):107. doi: 10.1038/s41408-022-00700-x (PMC9276717; doi:10.1038/s41408-022-00700-x)
Supplement: Supplementary file 3 — Supplementary Material [file 41408_2022_700_MOESM3_ESM.docx]

**Supplementary material**

**Statistical analysis**

In order to develop and validate a European scoring system for AML patients ≥ 70 years old treated with front line IC, we used two datasets. Data from the DATAML and PETHEMA registries (N=636) were used as a training set and data from the SAL registry (N=563) were used as an external validation set. The scoring system was based on OS (as the time between diagnosis and death or the last contact) censored at 5 years and included 6 candidate predictors (age, ECOG performance status (PS), white blood cell count (WBC) at diagnosis, secondary vs de novo AML, cytogenetic risk and NPM1/FLT3-ITD mutations). Previously published cut-offs in IC populations were used (1). The number of events (and non-events) per variable was > 20 for the training set (N=505 5-y deaths / 636 patients). As recommended, the validation dataset included at least 100 participants with the outcome (N=411/563) (2). In the training set, missing values were imputed using multiple imputations for PS, WBC at diagnosis and secondary vs de novo AML. Since there were many patients with missing cytogenetic or *NPM1*/FLT3-ITD mutations and in order to build a score (more adapted to actual clinical practice) which also included patients with missing cytogenetic or *NPM1/FLT3*-ITD mutations, missing results of cytogenetics and *NPM1*/*FLT3*-ITD mutations were included in the analyses as a dummy variable. We used STATA statistical software, version 17.0 (STATA Corp., College Station, TX) to conduct multiple imputations (mi commands) by chained equations (MICE). For binary variables imputation, logistic regression was used and for continuous variables imputation, a linear regression model was used. According to the proportion of 13% of incomplete cases, 15 imputed data sets were generated in the training population. After multiple imputation, the largest Fraction of Missing Information (FMI) was 14%, indicating that the number of imputed datasets created was adapted (3, 4). The imputation model included independent variables (candidate predictors) and the outcome (OS) together with auxiliary variables that predict missingness (based on the assumption that data were randomly missing) and/or that are associated with the missing variables. After multiple imputation, a multivariate Cox proportional hazards model (as recommended, without previous univariate analyses or stepwise selection) was used to assess β-coefficients of the survival predictors. All first-order interactions between predictors were tested and none were significant. The proportionality assumption was checked with Cox-Snell residuals and log-log plots (and was verified). A linear predictor (LP) based on the β-coefficients of the multivariate Cox model (after multiple imputation) was computed for all patients with a complete case in the training set. Moreover, to provide a simple tool for clinical practice, we developed score sheets. The formula (β-coefficient/abs(lowest β-coefficient)) rounded off to the nearest integer was used. Based on the predicted 5-year overall survival probability (S(t/LP)=S0(t)exp(β.LP)), three risk score categories were created (according to previously published survival probabilities from European data on DATAML, PETHEMA and SAL registries for IC (12%) and HMA (3%)) (1). To verify the internal validity of the LP, the R²D described by Royston and Sauerbrei (that is a measure of explained variation for survival models) was assessed together with measures of calibration and discrimination, in the training cohort. The calibration was deemed valid if the estimated parameter for LP was not statistically different from 1. The calibration was also checked by plotting the observed survival probability versus the predicted survival probability at five years (by quintiles of the predicted survival probability at five years). Discrimination was assessed using Harrell’s concordance index (C-index). This index estimates the proportion of all pairs of patients in which prediction and outcome are concordant. The C-index uses values from 0.5 (no discrimination) to 1.0 (perfect discrimination). The C-index for the Cox model with LP was corrected by subtracting the degree of over-optimism (obtained using 50 random bootstrap samplings with replacement). Discrimination was also assessed using Kaplan-Meier survival curves for the risk groups and estimating hazard ratios along with their 95% confidence interval (CI). Finally, discrimination was verified by assessing the effect of risk groups on other endpoints (complete remission (CR), Day-30 and Day-60 death (early death (ED)) and RFS). To verify the external validity, the R²D and C-index (for Cox model with the risk groups as factor) together with Kaplan-Meier survival curves for the risk groups were assessed in the external validation set. Finally, in the validation set, we compared the predictive performance of our risk groups to published prognostic indices. We estimated R²D, C-index, hazard ratios along with their 95% confidence interval using a Cox model with the risk groups and the false positive rate (rate of patients identified as high-risk in the subset of those who survived at 5 years). Tests that were two sided and p-values lower than 0.05 were considered significant. We described the patients’ characteristics using numbers and frequencies for qualitative data, and means with standard deviations (SD), medians with inter-quartile ranges (IQR) and range (minimum-maximum) for quantitative data. Comparisons between the patients’ characteristics were assessed using the Student’s t-test (or Mann-Whitney test when the distribution departed from normalcy or when homoscedasticity was rejected) for continuous variables, and the χ2-test (or Fisher’s exact test when small numbers were expected) for categorical variables. Kaplan-Meier curves were compared by the log-rank test. Statistical analyses were performed using STATA statistical software, version 17.0 (STATA Corp., College Station, TX).

1. Recher C, Rollig C, Berard E, Bertoli S, Dumas PY, Tavitian S, et al. Long-term survival after intensive chemotherapy or hypomethylating agents in AML patients aged 70 years and older: a large patient data set study from European registries. Leukemia. 2022;36(4):913-22.

2. PROBAST: A Tool to Assess Risk of Bias and Applicability of Prediction Model Studies: Explanation and Elaboration. Annals of Internal Medicine. 2019;170(1):W1-W33.

3. White IR, Royston P, Wood AM. Multiple imputation using chained equations: Issues and guidance for practice. Stat Med. 2011;30(4):377-99.

4. Bodner TE. What Improves with Increased Missing Data Imputations? Structural Equation Modeling: A Multidisciplinary Journal. 2008;15(4):651-75.
